# Heart Rate Variability reveals the fight between racially biased and politically correct behaviour

**DOI:** 10.1038/s41598-019-47888-w

**Published:** 2019-08-08

**Authors:** M. Di Palma, E. Arcangeli, D. Lattanzi, A. Gabbiadini, M. Gallucci, R. Cuppini, A. Minelli, M. Berlingeri

**Affiliations:** 1 Department of Humanistic Studies, University of Urbino Carlo Bo, Urbino, Italy; 2 Department of Biomolecular Sciences, University of Urbino Carlo Bo, Urbino, Italy; 3 Department of Psychology, University of Milano-Bicocca, Milano, Italy; 4 NeuroMi, Milan Centre for Neuroscience, Milan, Italy; 5 Center of Developmental Neuropsychology, Area vasta 1, ASUR Marche, Pesaro, Italy

**Keywords:** Social behaviour, Empathy, Human behaviour

## Abstract

In this study, we explored vagally-mediated heart rate variability (vmHRV) responses, a psychophysiological index of cognitive self-regulatory control, to map the dynamics associated with empathic responses for pain towards an out-group member. Accordingly, Caucasian participants were asked to judge the experience of African and Caucasian actors touched with either a neutral or a harmful stimulus. Results showed that (1) explicit judgment of pain intensity in African actors yielded higher rating score and (2) took longer time compared to Caucasian actors, (3) these behavioural outcomes were associated with a significant increment of RMSSD, Log-HF-HRV and HF-HRV n.u., (4) resting HF-HRV n.u. predicted the participants’ lag-time to judge painful stimulations delivered to African actors. Interestingly, these dynamics were associated with a measure of implicit racial attitudes and were, in part, abolished when participants performed a concurrent task during videos presentation. Taken together our results support the idea that a cognitive effort is needed to self-regulate our implicit attitude as predicted by the ‘Contrasting Forces Model’.

## Introduction

Empathy represents a cornerstone of human life^1^ as the ability to understand and share others’ emotions and mental states^2,3^ is at the basis of adaptive social interactions. For this reason, over the last decades clinical and cognitive psychologists, as well as neuroscientists, deeply investigated its behavioral, cognitive and neural correlates. One of the most accepted models in the literature suggests that empathy may be divided at least into two dissociable, although interacting, components: ‘affective empathy’ and ‘cognitive empathy’^4,5^. The former is regarded as a phylogenetically ancient automatic response that allows humans to vicariously experience the affective state of others by embodied simulation^6^. The latter process is closely tied to the mentalizing mechanism necessary to understand other’s perspective; this latter component may be affected by personal and contextual factors. From the theoretical point of view, cognitive empathy is strictly related to the concept of ‘perspective taking’^7^ and with the theory-of-mind^1^, as a consequence, while affective empathy may manifest itself also in the absence of ‘cognitive control’, cognitive empathy entails a certain degree of awareness^1,2^. In keeping with this model, various self-report measures (such as the Empathy Quotient^8^), as well as behavioral paradigms (such as the ‘Reading the Mind in the Eyes’ Test^9^) have supported the existence of the dichotomy between bottom-up affective and top-down cognitive processes during the unfolding of empathic responses. In line with the results of the self-report and behavioral studies, a number of neuroimaging evidence suggests that these two components can dissociate also at the neurofunctional level. Accordingly, affective empathy, as being responsible for the initial decoding phase of empathic processing, engages brain regions typically associated with motivational-affective dimensions, such as the insular and the anterior cingulate cortices, thalamus, amygdala, fusiform gyrus, somatosensory and motor cortices (see^2,3^ for details reviews). On the contrary, cognitive empathy engages brain regions typically associated with executive, higher-order functions, such as ventromedial, dorsolateral and dorsomedial prefrontal cortices, superior temporal gyrus, temporo-parietal junction, superior and inferior parietal lobules (see^1,2^ for details reviews).

Amidst the emotional states leading to empathic reactions, those related with pain have been most extensively explored. There is now compelling evidence that experiencing pain in first person and perceiving other’s pain share, at least in part, the same neurofunctional correlate, including brain regions involved in motivational-affective processing of pain and affective empathic response (e.g. anterior insular and anterior mid-cingulate cortices)^10,11^, as well as crucial nodes of the brain pain matrix involved in the experience or simulation of pain’s response (such as thalamic nuclei or primary and secondary somatosensory cortices)^12–14^. Many contextual interpersonal variables, such as those related to the in-group/out-group social categorizations^15^, can affect empathic processing in humans; in particular, the existence of racially-biased empathic responses for pain has been evidenced and broadly characterized in electrophysiological^16^, Evoked Response Potentials^17^, Transcranial Magnetic Stimulation (TMS)^18^ and functional Magnetic Resonance Imaging (fMRI) studies^19–22^. Two recent fMRI studies^19,22^ have reported a greater activation of the cerebral pain matrix, especially the cingulate and insular cortices, when participants observed an actor of their same race (in-group) receiving a harmful stimulus than when observing actors of a different race (out-group); this neural signature of implicit racial biases has been called ‘Differential Empathic Activation for Race (DEAR) effect’^20^. In spite of well-documented DEAR effect, different works have pointed out that, on average, people tend to judge the out-group actors’ experience to harmful stimuli as painful as the one administered to in-group actors^19–21^; this suggests that, in order to conform to social norms, people may exhibit controlled, egalitarian behaviors toward out-group members, thus self-regulating opposite implicit and embodied empathic reactions^23^. Along with this line of reasoning, using a fMRI paradigm, Belingeri *et al*.^20^ introduced a dual-route model named ‘Contrasting Forces’. According to this model the empathic responses for pain would be shaped by the interaction between two dissociable empathic components, one associated with affective empathy, and as a consequence, more automatic and embodied, one associated with cognitive empathy and, as a consequence, more controlled and ‘strategic’. The former is depicted by what the authors called in-group DEAR effect (an early preferential activation of the pain matrix regions when in-group actors are delivered with harmful stimuli), the latter is represented by the so-called out-group DEAR effect. This effect would be responsible for the manifestation of overtly (behavioral) egalitarian responses even in the presence of implicit racial negative attitudes (as measured by the Implicit Association Test; IAT^24^) and it is supposed to compensate the lack of a prompt embodied simulation when observing an out-group’s actor experiencing a painful situation. From the neurofunctional point of view, the out-group DEAR effect seems to be associated with the activity of the dorsolateral prefrontal cortex: an area typically associated with cognitive empathy^25^, self-regulation^26^ and strategic and controlled behavior^27^. From a behavioral point of view, to judge how painful an experience was for an out-group actor takes approximately 100 milliseconds longer than judging a similar situation in which is involved an in-group one^20^. In other words, we need time to self-regulate and counterbalance our biases in order to manifest an overt behavioral response in line with social rules. If this is the case, then a concurrent task capable of subtracting resources to the main task, i.e. the empathic judgment task, should prevent from the cognitive reappraisal of the empathic situation and let the implicit attitudes emerge. This hypothesis would be in line with the behavioral results by Yzerbyt *et al*.^28^ showing that a reduction of cognitive control is capable to reveal the existing racially biased stereotypes. Flexibly adapting our behavioral strategies to the social context requires the continuous assistance of multi-systemic, integrated body responses that are dynamically regulated by visceral activity and deployment of metabolic resources^29^ for the sake of the organism’s homeostasis and fitness. According to the ‘Neurovisceral Integration Model’ proposed by Thayer and Lane^30,31^, a network of reciprocally connected brain regions, the ‘central autonomic network’ (CAN)^32^, would work as a ‘super-system’ that integrates the activity of perceptual, motor, interoceptive and memory systems into adaptive responses, dynamically coupling central neural activity with peripheral autonomic drive on multiple physiological systems^29^. In particular, the vagally-mediated heart rate variability (vmHRV), an index of parasympathetically-induced changes in consecutive heart beats^33^, is regarded as a promising biomarker of CAN activity in the service of behavioral flexibility^34–36^. Indeed, resting vmHRV is closely related to the effective engagement of prefrontal-subcortical inhibitory circuits implicated in the self-regulatory effort of emotional and cognitive processes^37–39^. Empirical evidence suggests that individuals with high resting vmHRV are more efficient in regulating both emotional and cognitive processes during simulation and interpretation of the respective states^40–44^, moreover, irrespectively from the resting vmHRV level, emotion suppression and reappraisal have been associated with vmHRV enhancement^43,45–47^. In spite of the well-documented link between vmHRV and cognitive and emotion regulation, only a few studies have investigated the association between vmHRV and the empathy for pain^48–50^, however, to date it has never been explored whether the vmHRV responses detected during empathic conditions can be moderated by social factors such as the in-group/out-group dichotomy. This is exactly the aim of this study. By taking the assumption that vmHRV changes are typically associated with reappraisal and emotion regulation processes, we aim at investigating whether self-regulatory effort while explicitly judging the level of pain inflicted to an out-group member (out-group DEAR effect) is associated with specific vmHRV changes and whether these changes can be influenced by the level of implicit racial bias of each single subject. As a consequence, participants were presented with a series of video clips showing either Caucasian or African actors stimulated with either a neutral, or a harmful stimulus, and with a series of implicit and explicit measure of racial bias. We recorded the vmHRV signal in two different experimental settings: namely, a single-task and a classic dual-task condition to test this specific set of hypotheses: (1) in line with Berlingeri *et al*.^20^ longer reaction times are needed to judge a harmful stimulus delivered to an out-group member as painful as the same stimulus delivered to an in-group member in a single-task condition (this would indicate that greater self-regulatory effort is needed to conform to social-rules in the presence of an implicit negative attitudes, on average), (2) in the single-task condition, greater self-regulatory effort should be associated with higher vmHRV changes^43,45,47^, (3) the higher the capacity to self-regulate our inner emotional state and to flexibly adapt our behavioral responses to social norms, the higher the level of vmHRV at rest, in line with several works^34,38,39,42,43,45,51,52^ suggestion, (4) the vmHRV differences between the in-group and the out-group painful stimulations should correlate with the subjective level of implicit racial bias estimated by means of the IAT^24^ in line with Forgiarini *et al*.^16^ in a single-task condition; finally, (5) in line with Yzerbty *et al*.^28^ we assume that all the dynamics envisaged for the single-task condition, would be affected by a concurrent cognitive task capable of stressing the executive system.

## Results

### Participants and behavioral scales

Male and female participants were matched both for age [*t*_(46)_ = 0.218, *p* = 0.8283; see Table 1] and, for the Body Mass Index [*t*_(46)_ = 1.893, *p* = 0.0646; see Table 1]. A series of further Student’s *t*-test also revealed no differences between male and female participants in Resting Root Mean square of Successive Differences - RMMSD [*t*_(46)_ = 0.261, *p* = 0.7949; see Table 1], Resting natural Log transformed High-Frequency power - Log-HF-HRV [*t*_(46)_ = 0.377, *p* = 0.7079; see Table 1], Resting High-Frequency power normalized units - HF-HRV n.u [*t*_(46)_ = 0.276, *p* = 0.7837; see Table 1], Resting peak High-Frequency values – pHF-HRV [*t*_(46)_ = 1.144, *p* = 0.2585; see Table 1] and Symphatovagal balance Index – SVI [*t*_(46)_ = 0.223, *p* = 0.8227; see Table 1]. On the other side, results also revealed no differences between male and female participants in Resting natural Log-transformed Low-Frequency power and Resting Low-Frequency power normalized units (results not shown, for details see Supplementary Information; S-Table 4). Regarding the implicit association measures (IAT^24^), overall our sample obtained a mean IAT D score of 0.80 (standard error of mean = 0.04; one sample Student’s *t*-test (H_0_: μ = 0): *t*_(47)_ = 19.84, *p* < 0.001), which indicates a significant implicit association between negative words and Africans pictures. The descriptive statistics of the behavioral scales, Internal Motivation Scale and External Motivation Scale score, Scale for Ethnocultural Empathy score, Subtle and Blatant Prejudice score and Trait Empathy Scale score are reported in Table 2, it is worth noting that the sample included in this study behaves, on average, similarly to the participants included in previous studies^16,18,20^.

**Table 1:** Participants’ characteristics.

	Male participants				Female participants				Test statistic	
	Mean	SEM	Min	Max	Mean	SEM	Min	Max	*t*, *df*	*p*
Age	24	0.92	20	35	24	1.049	19	35	0.218, 46	0.8283
Body Mass Index (kg/m^2^)	23.12	0.53	18.46	29.32	21.77	0.44	18.03	25.87	1.893, 46	0.0646
**Heart rate variability**										
Resting RMSSD	41.55	2.47	20.93	70.2	40.66	2.27	24.56	58.39	0.261, 46	0.7949
Resting Log-HF-HRV	6.69	0.16	4.97	8.55	6.60	0.14	5.24	7.82	0.377,46	0.7079
Resting HF-HRV n.u.	31.44	1.67	19.34	53.47	32.15	1.93	15.64	47	0.276, 46	0.7837
Resting pHF-HRV	0.17	0.01	0.15	0.28	0.19	0.01	0.15	0.32	1.144, 46	0.2585
Resting SVI	2.36	0.17	0.87	4.17	2.29	0.28	1.06	6.57	0.223, 46	0.8227

Resting RMSSD = Root mean square of successive differences from HR resting recordings (5-min), Resting Log-HF-HRV = natural log transformed high-frequency heart rate variability power from HR resting recordings (5-min), Resting HF-HRV n.u. = high-frequency heart rate variability power normalized units from HR resting recordings (5-min), Resting pHF-HRV = peak high-frequency heart rate variability values from HR resting recordings (5-min), Resting SVI = Sympathovagal balance index from HR resting recordings (5-min).

**Table 2:** IMS = Internal Motivation Scale^66^, EMS = External Motivation Scale^66^, EES = Scale of Ethnocultural Empathy^67^, TES = Trait Empathy Scale^70^.

	Median	Min	Max	one-sample Wilcoxon *p*
**Self-report measures**				
IMS	6.2	1	7	0.0001**
EMS	3.3	1	5.8	0.0001**
EES	4.4	3.2	5.2	0.0001**
Subtle Prejudice Scale	4.1	2.4	5.5	0.0001**
Blatant Prejudice Scale	2.5	1.4	5.2	0.0001**
TES	4.9	3.4	6.1	0.0001**

### Behavioral results

**Figure 1:**
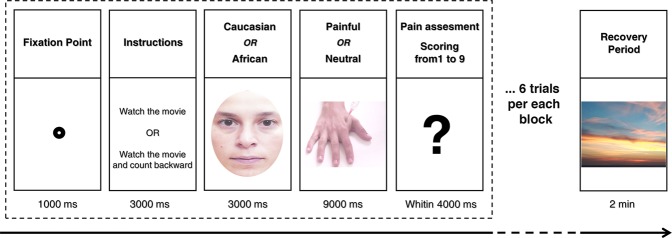
Example of a trial within one of the experimental blocks. Each block began with a fixation point for 1000 ms, followed by the instruction(in the single-task condition: ‘watch the movie’, in the dual-task condition ‘watch the movie and count backward’); then the video clip started with a frame showing the actor’s face holding a neutral expression. By the 3th second, the camera zoomed in on the actor’s hand, which was then touched alternatively by a rubber eraser (neutral stimulus), or by a needle (harmful stimulus), then within 4000 ms, participants were instructed to explicitly judge the painful experience of the actor using a Likert scale from 1 (not painful at all) to 9 (highly painful) by pressing an appropriate key on the number pad with their dominant hand. This sequence (within the dashed-line rectangle) was repeated six times per block; the experimental blocks were separated by a 2-minute interval during which participants were asked to watch a relaxing video clip.

Based on previous findings by Berlingeri *et al*.^20^, we expected that (i) participants would judge the African actors’ harmful stimuli as painful as those administered to the Caucasian actors (see Methods and Fig. 1), (ii) they would need more time to produce an explicit response in line with social norms. To test this hypothesis, the explicit empathic responses and the RTs collected during both single and dual experimental task were analyzed by means of general linear mixed-effects model (GLMM; see Supplementary Information for further details).

**Figure 2:**
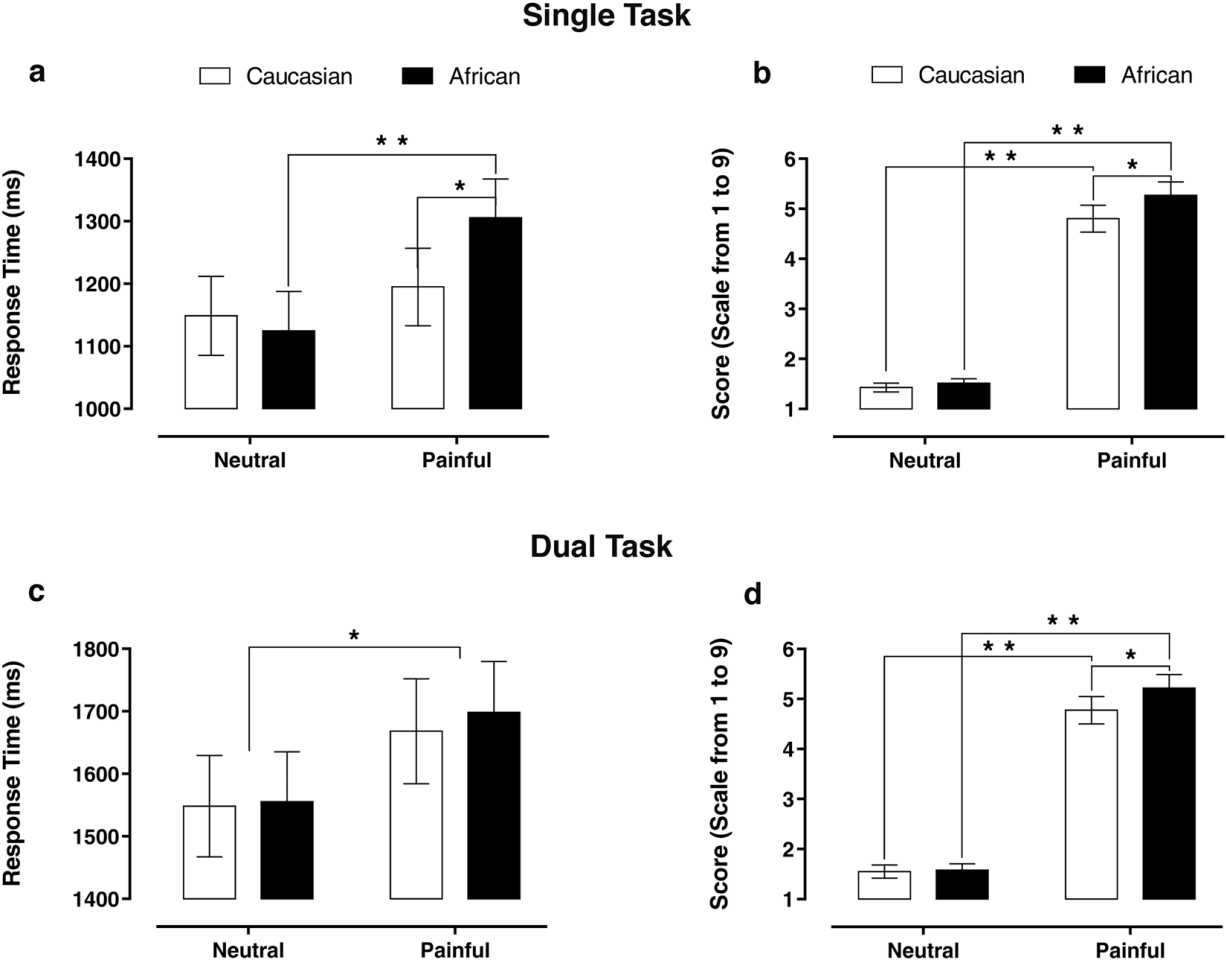
Behavioral data collected during the experimental task. The bar-graphs in the left panel of the figure represent the reaction times associated with explicit judgments in both single (**a**) and dual (**c**) task conditions. The bar-graphs on the right panel of the figure represent the score value of the explicit judgments for the Caucasians and Africans actors in both single (**b**) and dual (**d**) task conditions. Values are expressed as mean ± SEM; *p < 0.05; **p < 0.001.

Explicit behavioural scores (Scores; measured by a Likert scale from 1 to 9): in the single-task condition, results showed a significant *main effect of type of stimuli* (*χ*^*2*^_(1,1078)_ = 205.84; *p* < 0.001), a significant *main effect of race* (*χ*^*2*^_(1,1078)_ = 17.34; *p* < 0.001) and a significant *type of stimuli-by-race interaction effect* (*χ*^*2*^_(1,1078)_ = 8.50; *p* = 0.003). We further explored this interaction, and we observed that participants attributed higher explicit scores to the Africans’ experience with harmful stimuli when compared with scores elicited by Caucasian actors (*χ*^*2*^_(1,1078)_ = 24.90; *p*_*FDR-corrected*_ < 0.001; see Fig. 2b).

Similarly, in the dual-task condition, results showed a significant *main effect of type of stimuli* (*χ*^*2*^_(1,976)_ = 164.82; *p* < 0.001), a significant *main effect of race* (*χ*^*2*^_(1,976)_ = 9.22; *p* = 0.002) and a significant *type of stimuli-by-race interaction effect* (*χ*^*2*^_(1,976)_ = 8.22; *p* = 0.004). This interaction was further explored, and we observed that, even with a concurrent task during videos presentation, higher explicit scores were attributed to the Africans’ experience with harmful stimuli (*χ*^*2*^_(1,976)_ = 17.37; *p*_*FDR-corrected*_ < 0.001; see Fig. 2d).

RTs: in the single-task condition results showed a significant *main effect of type of stimuli* (*χ*^*2*^_(1,1047)_ = 7.23; *p* = 0.007) and a significant *type of stimuli-by-race interaction effect* (*χ*^*2*^_(1,1047)_ = 5.18; *p* = 0.022). Accordingly, there was a significant difference between the RTs collected for Caucasian and African actors in the painful condition (*χ*^*2*^_(1,1047)_ = 6.78; *p*_*FDR-corrected*_ = 0.018) and a significant type of stimuli effect for Africans actors only (*χ*^*2*^_(1,1047)_ = 12.31; *p*_*FDR-corrected*_ < 0.001; see Fig. 2a). On the other hand, in the dual-task condition, only a significant *main effect of type of stimuli* (*χ*^*2*^_(1,970)_ = 4.12; *p* = 0.04) was observed (see Fig. 2c).

### Psychophysiological results

**Figure 3:**
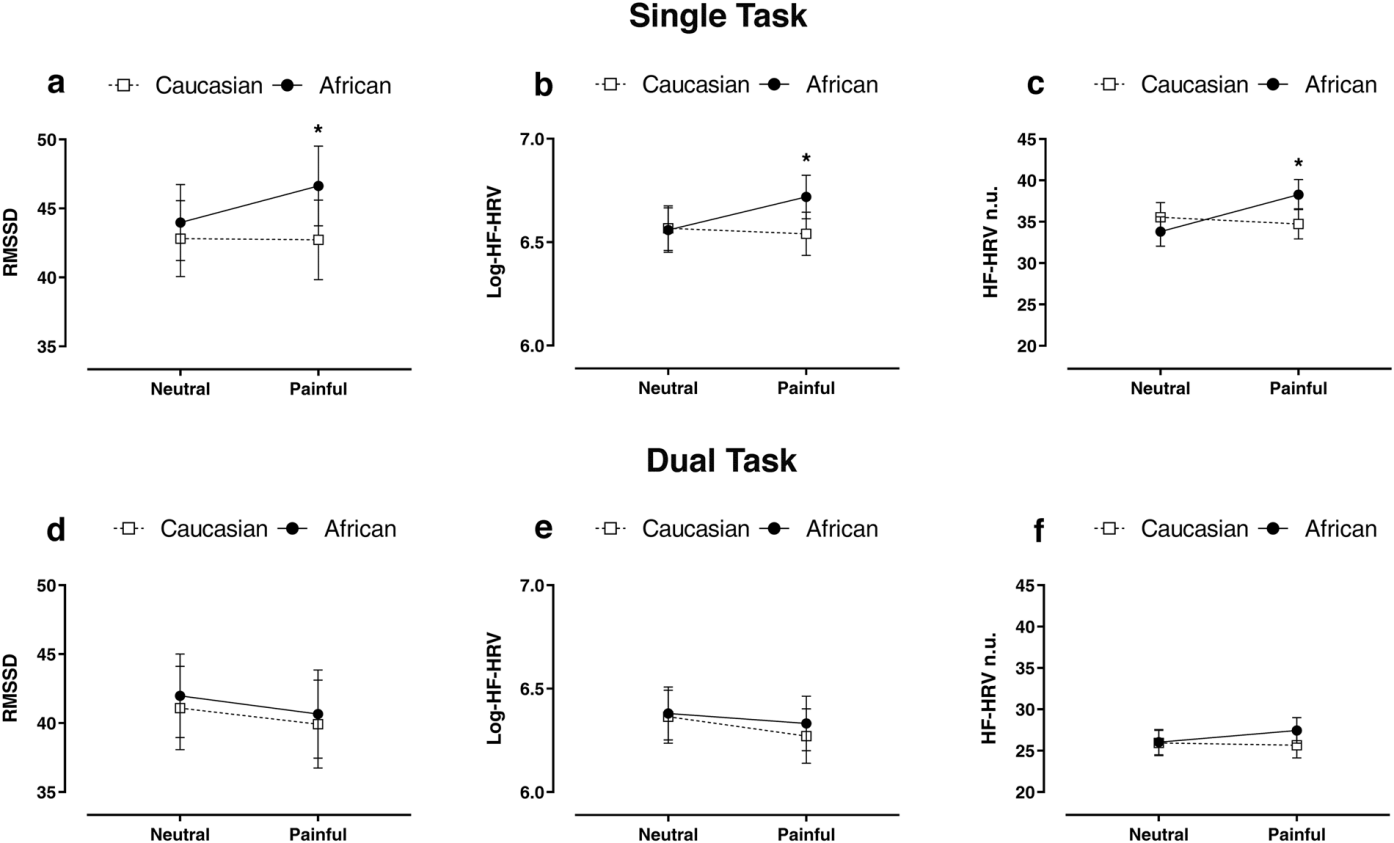
vmHRV differences collected during the experimental task. The line-plot graphs represent the RMSSD, Log-HF-HRV and HF-HRV n.u. as a function of stimulus type and actor’s race in both the single (**a**–**c**) and dual (**d**–**f**) task conditions. Values are expressed as mean ± SEM; *p < 0.05.

**Figure 4:**
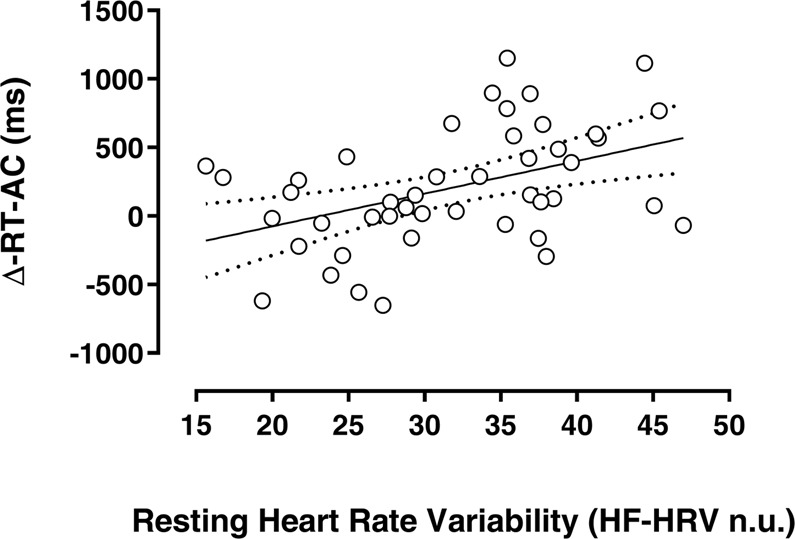
Linear regression between Resting HF-HRV n.u. and Δ-RT-AC (out-group vs in-group) in painful stimulations. The solid line indicates the best linear model and the dashed lines indicate the 95% confidence intervals.

In accordance with previous findings^43,45,47^, we expected that the greater self-regulatory effort needed to explicitly judge the painful experience of African actors should be associated with a higher vmHRV. To test this hypothesis, all the vmHRV measures calculated in both single and dual experimental task, namely, RMSSD (as time-domain measure of vmHRV), HF-HRV and HF-HRV n.u. (as frequency-domain measures of vmHRV), were analysed by means of a GLMM (see Supplementary Information for further details about the model’s syntax). RMSSD: in the single-task condition, results showed a significant *main effect of race* (*χ*^*2*^_(1,192)_ = 14.30; *p* < 0.001) and a significant type of *stimuli-by-race interaction effect* (*χ*^*2*^_(1,192)_ = 3.70; *p* = 0.050). This interaction was further explored, and we observed a higher RMSSD referred to Africans painful stimulation (*χ*^*2*^_(1,192)_ = 16.44; *p*_*FDR-corrected*_ < 0.001; see Fig. 3a). Prior to analyse the frequency-domain measures of vmHRV, the peak of HF-HRV (pHF-HRV) a measure of respiratory frequency, was explored in both single and dual-task to control for potential bias induced by respiratory frequency during the experimental tasks^33,53^. In both single and dual-task experimental conditions, pHF-HRV average values were in the range of 0.15–0.23 Hz and no significant *main effects* were observed (results not shown, for details see Supplementary Information; S-Fig. 4), thus showing that respiratory frequency did not affect the frequency-domain measures of vmHRV^54^.

**Figure 5:**
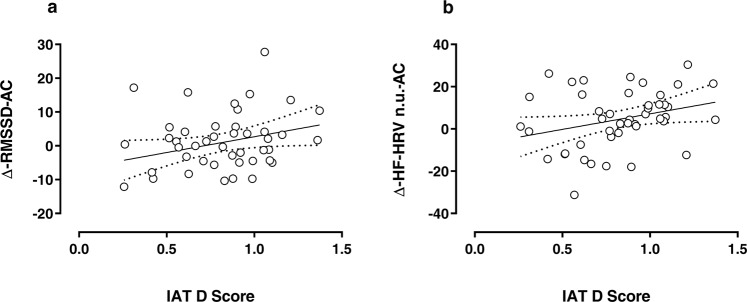
Linear regression between IAT D Score and Δ-RMSSD-AC (**a**) and Δ-HF-HRV n.u.-AC (**b**). The solid line indicates the best linear model and the dashed lines indicate the 95% confidence intervals.

Frequency-domain measures of vmHRV: similarly, to the pattern of results that emerged from RMSSD, even with HF-HRV in the single-task condition we found a significant *main effect of race* (*χ*^*2*^_(1,192)_ = 3.70; *p* = 0.05), as well as, a significant type of *stimuli-by-race interaction effect* (*χ*^*2*^_(1,192)_ = 4.44; *p* = 0.035). In particular, participants showed higher HF-HRV referred to Africans painful stimulation (*χ*^*2*^_(1,192)_ = 8.12; *p*_*FDR-corrected*_ = 0.008) and a significant type of stimuli effect for African actors (*χ*^*2*^_(1,192)_ = 5.74; *p*_*FDR-corrected*_ = 0.032; see Fig. 3b). HF-HRV n.u.: in the single-task condition, we found a significant *type of stimuli-by-race interaction effect* (*χ*^*2*^_(1,192)_ = 5.70; *p* = 0.016) with a higher HF-HRV n.u. when African actors were touched with a harmful stimulus as compared to the Caucasians (*χ*^*2*^_(1,192)_ = 5.15; *p*_*FDR-corrected*_ = 0.046) and a significant type of stimuli effect specific for Africans actors (*χ*^*2*^_(1,192)_ = 7.43; *p*_*FDR-corrected*_ = 0.012; see Fig. 3c). Notwithstanding, the results of the post-hoc comparisons survived the false discovery rate correction, here it is worth noting that the lack of a complete cross-over interaction suggests that this latter result should be taken with a grain of salt. On the other hand, in the dual-task condition, no significant *main effects* were observed in all vmHRV measures (see Fig. 3d–f); the pattern of results emerged from Low Frequency-HRV (LF-HRV) analyses mirrored the one obtained with HF-HRV (for details on LF-HRV measures, see Supplementary Information S-Fig. 5).

### Resting HRV and reaction times

According to recent works^34,38,39,42,43,45,51,52^, the higher the level of vmHRV at rest, the higher the capacity to self-regulate our inner emotional state flexibly adapting our behavioural responses to social norms. A self-regulating effort is reflected by lag-time, according to the ‘Contrasting Forces Model’^20^, to explicitly judge a painful experience of an out-group member compared to an in-group member, and this lag-time is associated with an increase of the activity of the prefrontal cortex: an area typically associated with self-regulation^26^, strategic and controlled behavior^27^. Collectively these findings prompted us to hypothesise that the delay in rating the painful experience of out-group actors could be likely related to the involvement of prefrontal-subcortical inhibitory circuits implicated in the self-regulatory effort of emotional and cognitive processes^37,39,51^. To explicitly test this hypothesis, the relationship between resting vmHRV and Δ-RT-AC was explored by means of a General Linear Model (GLM) in the single-task only. The results of the GLM showed that the resting HF-HRV n.u. is a significant predictor of Δ-RT-AC (*b* = 20.92, *t*_(43)_ = 2.79, *p* = 0.005; for more details see Supplementary Information S-Fig. 2), thus with higher HF-HRV n.u., we are expecting a longer delay in judging the painful experience for Africans as compared with Caucasians (see Fig. 4). RMSSD and Log-HF-HRV did not show any significant linear relationship to Δ-RT-AC.

### HRV changes and implicit racial bias

Based on the findings by Forgiarini *et al*.^16^, we expected that in a single-task condition vmHRV differences between the out-group vs. in-group painful stimulations should correlate with the subjective level of implicit racial bias (IAT). To test this hypothesis, the association between IAT D scores and Δ-vmHRV-AC values in single-task condition was explored by means of a GLM. The results showed that the IAT D score is a significant predictor of both Δ-RMSSD-AC (*b* = 9.34, *t*_(44)_ = 3.91, *p* = 0.04; see Fig. 5a;) and Δ-HF-HRV n.u.-AC (*b* = 14.72, *t*_(44)_ = 4.00, *p* = 0.04; see Fig. 5b; for more details see Supplementary Information S-Fig. 3), thus for every increase in the IAT D score, we are expecting an increase in the Δ-RMSSD- and Δ-HF-HRV n.u.-AC measures (see Fig. 5).

## Discussion

The purpose of the present study was to investigate whether self-regulatory effort needed to explicitly judge the pain experienced by an out-group member (out-group DEAR effect) is associated with specific vagally-mediated HRV (vmHRV) changes and whether these changes can be affected by the subjective level of implicit racial attitude.

From a behavioral point of view, our data confirm and extend previous findings by Berlingeri *et al*.^20^. In particular, we replicate that, on average, significant longer RTs (approximately 100 ms) are needed to explicitly judge the painful experience stimulation of African than Caucasian actors (see Fig. 2a). Moreover, by using a nine-digit-Likert scale providing a finer grain characterization (thus higher resolution) of the out-group DEAR effect, we extend previous findings showing that significant higher explicit scores were attributed to harmful stimuli experienced by Africans than Caucasians actors (see Fig. 2b). Thus, consistent with previous findings^20^ our data confirm that in the single-task condition (i.e. in absence of additional cognitive load) participants needed more time to explicitly judge the painful experience of an out-group actor, and for the first time we show that participants, in order to adapt their behavior to social norms, judge the level of pain experienced by out-group actor higher than the one experiences by in-group actors (see Fig. 2a,b). This latter finding is particularly intriguing from a social point of view since it seems to suggest that the social norms and social desirability can elicit such a pressure to promote something more than an egalitarian response, i.e. an overcorrection of our explicit empathic response that, paradoxically, may become unfair toward in-group members, something, in a way, similar to the neurocognitive processes described in the ‘altruistic behavior’^55^.

From a psychophysiological point of view, our data show that the out-group DEAR effect is associated with specific vmHRV changes measured in both time and frequency domains. In particular, we found a significant, on average, enhancement of RMSSD, HF-HRV and HF-HRV n.u. associated with the task of giving an explicit judgment of pain experienced by African actors (see Fig. 3a–c). According to the ‘Neurovisceral Integration Model’ proposed by Thayer and Lane^30,31^, vmHRV enhancement would reflect the engagement of prefrontal-subcortical inhibitory circuits, the core circuitry of the ‘central autonomic network’, which play a critical role in self-regulatory function by dynamically coupling central neural activity with peripheral autonomic drives in the service of behavioral flexibility^56^. In this regard, vmHRV enhancement was observed in several behavioral tasks (e.g. food temptation task^43^, emotional suppression or emotion reappraisal task^45^) which demanded self-regulatory effort. Our data extend these previous findings by showing for the first time, in a model of empathic reaction for pain stimuli, that vmHRV enhancement can be regarded as a reliable psychophysiological marker reflecting the cognitive self-regulatory effort needed to mitigate our negative attitudes and to adapt our behavior to social norms in an empathy for pain task. Present findings are in line with previous neurofunctional findings revealing that the out-group DEAR effect is associated with the activity of a cortical area involved in self-regulation and cognitive reappraisal process as the dorsolateral prefrontal cortex^20^.

Interestingly, the administration of a concurrent task, imposing high cognitive load on the executive prefrontal system (i.e. dual-task condition), was able to abolish the differences observed in single-task condition in RTs (see Fig. 2c) and psychophysiological outcomes (see Fig. 3d–f). These findings, in accordance with Yzerbty *et al*.^28^, further support that a prefrontal top-down self-regulatory drive is needed to mitigate our negative attitudes and to manifest an overt behavioral response toward out-group members in line with social rules. However, it worth noting that, even in dual-task condition, our participants showed an overcorrection toward out-group members (see Fig. 2d). This finding supports the assumption that social norms and education can shape our neurocognitive system to such an extent that the inner racial-bias may be counterbalanced also in a relatively stressful condition, and that a higher cognitive load, more stressful than that used in the present study, is needed to possibly unveil negative attitudes, something that in real-words conditions can actually happen.

Previous psychophysiological studies showed that individuals with high resting vmHRV are more efficient in regulating both emotional and cognitive processes during explicit emotion regulation tasks^38,39,51^. Our findings are partially in line with this evidence. In fact, we showed that resting HF-HRV n.u. is a predictor of the out-group DEAR effect, i.e. the higher was participant’s resting HF-HRV n.u., the longer was the lag-time to judge the pain experienced by African vs. Caucasian actors explicitly. However, resting RMSSD and HF-HRV failed to predict the out-group DEAR effect. Besides differences in the experimental task used in the present study (empathic responses for pain task vs explicit emotional regulation task), this apparent discrepancy may be related to the integrated nature of the HF-HRV n.u., representing the HF-HRV values in proportion to the total power (thus including LF-HRV) minus the VLF components^54^. Collectively, present evidence suggests, that resting HF-HRV n.u. may be adopted as a reliable predictor of the attitude to cognitive reappraisal in the serve of counterbalancing the inner biases during empathic responses for pain toward out-group members (Fig. 4).

Finally, we analyzed the relationship between vmHRV signatures of the out-group DEAR effect (an explicit explanation of how this effect was computed can be found in the methods section) and the subjective level of implicit racial bias (IAT). Our participants showed, overall, a significant implicit association between negative words and Africans’ pictures (evaluated by means of IAT D score) to an extent that was comparable with previous reports^16,20^. Interestingly, we found that IAT D scores were positively associated with RMSSD and HF-HRV n.u. enhancement when judging out-group harmful stimuli (with HF-HRV showing a positive trend but not reaching the significance see Fig. 5). Therefore, we can conclude that the IAT D scores of the participants significantly predict the vagally-mediated racially-driven empathic responses for pain. This latter finding may be seen as a complement of previous findings by Forgiarini *et al*.^16^ showing that, when watching harmful stimuli inflicted to out-group members, IAT D scores were negatively correlated with the amplitude of skin conductance response, a sympathetic psychophysiological index of the autonomic response in empathic reactions for pain.

Of course, the present study is not free from limitations. Firstly, future studies should better characterise participants empathy for pain and vicarious pain by adding, for example, a pain-specific empathy questionnaire like the Empathy for Pain Scale^57^ to complement the results obtained with the Trait Empathy Scale. In addition, we are aware that gender-related differences in vmHRV parameters have been reported in literature^58^; in a recent meta-analysis, Koenig and Thayer^58^ reported that females, as compared to males, showed greater resting HF-HRV power. However, in our sample, we failed to observe any major differences in resting HF-HRV between males and females (see Table 1). Sex-related differences tend to increase with advancing age^58^; therefore, we can hypothesize that the similarity of resting vmHRV values between female and male participants observed in our study might be due, at least in part, to the relatively young age of the participants. Concerning the abolishment of vmHRV differences in the dual-task setting, it cannot be ruled out, at least in part, that this could reflect a cardiac involvement in a stress response. Indeed, it is worth mentioning that a mental arithmetic task could evoke a stress response, as reported by several studies (for a detailed review see^59^, where a variety of cognitive tasks, including mental arithmetical tests, especially when performed under social evaluation, evoke changes in neuro-vegetative balance typical of acute stress responses. Finally, future studies should also consider a possible correlation between IAT D scores and measures of participants’ executive function.

In conclusion, here we show for the first time that vmHRV, a well-known psychophysiological index of prefrontal top-down executive/cognitive control, is a reliable index of the self-regulatory effort needed to adapt our behavior to social norms when we are required to empathize with the pain experienced by an out-group member. Accordingly, we gave the first psychophysiological picture of the contrasting forces model in action to suggest that despite the well-documented existence of implicit racially-biased empathic responses for pain^16–22^, we are able to exhibit controlled behaviors toward out-group members by cognitive self-regulating our-selves^19–21^. Interestingly, our findings could be considered also like the ‘other side of the coin’ of the results reported in the study by Tamir and Mitchell^60^ in which it has been showed that the anchoring and adjustment process is specifically engaged for in-group members, and not for out-group members where, as shown by our findings, a self-regulation process seems to be implicated.

This is an important empirical finding with a promising transferable value in the field of applied psychology and in supporting social and educational programs capable of promoting racial integration in modern societies.

## Methods

### Participants

Participants were recruited according to the following criteria: aged between 18 and 35 years, native Italian speakers and Caucasians. According to established recommendations for using vmHRV in psychophysiological research^54^, an ad-hoc created questionnaire was administered to all participants at the beginning of the experimental session. The ad-hoc created questionnaire included items associated with the following variables: gender, smoking, habitual levels of alcohol consumption, weight and height, cardioactive and psychotropic medications, oral contraceptive intake for female participants, follow a normal sleep routine the day before the experiment, no intensive physical training the day before the experiment, no meal the last 2 h before the experiment, no coffee – or caffeinated drinks or tea in the 2 h before the experiment and no alcohol consumption for 24 h prior the experiment (see Supplementary Information for further details about the ad-hoc created questionnaire; Fig. S-1).

In keeping with an *a priori* analysis performed with G*Power3^61^, we estimated to recruit 40 participants to be able to detect large effects size (*f* = 0.72, 1-β = 0.95, α = 0.01) about the association between specific vmHRV changes and self-regulatory effort while explicitly judging the severity of pain inflicted to an out-group member, namely, out-group DEAR effect. This analysis was performed by taking into account the results reported in the paper by Forgiarini *et al*.^16^, i.e. by considering the study that is closer to ours in terms of experimental paradigm, stimuli, and collected measures. Thus, taking into account potential outliers in the baseline condition, fifty-two young and healthy Caucasian participants were recruited among undergraduate university students and young workers. Of the fifty-two participants that were included in the study, we excluded data from four (females) who displayed high resting stress level values (assessed as Sympathovagal Balance Index^62–64^) during the 5-min resting HR recordings (for more details see the Behavioral and physiological analyses section). Consequently, the data of forty-eight participants, 27 males and 21 females, were considered in the analyses (see Table 1). All participants provided their written informed consent to take part in the study that was approved by the Ethics Board of the University of Urbino – Carlo Bo, and carried out in accordance with the Declaration of Helsinki^65^.

### Behavioural scales

Once arriving at the Neuropsychology and Psychometrics laboratory of the University of Urbino, all participants were individually tested in a quiet and dimly-lit room (temperature 22–23 °C). Before experimental task and vmHRV recording, participants completed the Internal Motivation to Respond Without Prejudice Scale (IMS)^66^, the Scale for Ethnocultural Empathy (EES)^67^, the Subtle and Blatant Prejudice Scales^68,69^ and the Trait Empathy Scale (TES)^70^. Participants also completed a race (Caucasian and African) Implicit Association Test (IAT) to assess the implicit racial bias^24^. In particular, in each trial of the race IAT, participants categorized a stimulus from one of the following four categories: a picture of Caucasian man, a picture of an African man, a positive word (e.g. joy, peace, love, good), or negative word (e.g. agony, war, pain, evil). The stimuli were organized into seven blocks. The critical blocks consisted of 24 trials. The IAT scores were calculated using an ad-hoc-created R routine to obtain the D score as described in Greenwald *et al*.^71^. The D scores were coded in the direction of the association between positive words and Caucasian targets; as a consequence, the higher the score, the higher the association between positive concepts and the Caucasian race (as well as the stronger the association between negative concepts and African actors).

### Experimental task stimuli

Stimuli were 12-second-long video clips depicting male or female actors (six Caucasian, 3 males and 3 females; six African, 3 males and 3 females) touched on their hand with either painful or neutral stimulus. Each video clip began with a frame showing the actor’s face holding a neutral expression. By the 3th second, the camera zoomed in on the actor’s hand, which was then touched by a Caucasian experimenter by either a neutral stimulus (eraser, the top of a pencil, cotton bud), or a harmful stimulus (needle, dagger, nail), followed by a 4-second still image of the hand/tool interaction. Stimuli were presented on a 60-Hz Samsung S22F350FH, with a screen diagonal of 22 in. connected to a DELL Precision Tower 5810 pc running the Windows 10 operating system.

### Experimental task procedure

Participants were asked to utilize the bathroom to control for the effects of bladder filling and gastric distension on vmHRV^72^. Participants were then seated in a comfortable chair in front of the computer monitor where the stimuli were displayed and they were advised to keep an appropriate posture for reaching without postural changes the number pad. Participants then were prepared for a 5-min of resting heart rate (HR) recording during which they were instructed to breathe spontaneously keeping their eyes open. The experimental task was divided into two different experimental settings: single-task (participants had to watch the video clip and explicitly judge the severity of pain) and dual-task (participants had to watch the video clip and explicitly judge the severity of pain while performing backward counting by 7 from a randomly generated three-digit number, e.g. ‘serially subtracting 7 from 305′). Forty-eight video clips (24 for single and 24 for dual-task) were presented in 8 separate blocks (4 blocks for each experimental setting, i.e. single-task vs dual-task), each block included six trials. Within each experimental setting, a full factorial design was implemented by combing the categorical factor ‘race’ (Caucasian vs African) with the factor stimulus-type (harmful vs neutral) obtaining, as a consequence, 4 experimental conditions: Caucasian actors touched with a harmful stimulus; Caucasian actors touched with a neutral stimulus; African actors touched with harmful stimulus and African actors touched with neutral stimulus. Each trial began with a fixation point for 1000 ms, followed by the instruction identifying the experimental setting (‘watch the movie’ or ‘watch the movie and count backward by 7′) for 3000 ms; then the video clip started and at the end of the video-clip participants were required to explicitly judge how painful was the actor’s experience using a Likert scale from 1 (not painful at all) to 9 (highly painful) by pressing an appropriate key on the number pad with their dominant hand. Participants were prompted to respond as quickly as possible, only reaction times (RTs) < 4000 ms were recorded. The 8 experimental blocks were separated by a 2-minutes interval during which participants were asked to watch a relaxing video clip (see Fig. 1), this was done in order to wash out any possible experimental effect between following blocks. The trials order within each block, as well as the order of each block within the experimental condition, was randomly assigned using the Python-based experimenter builder OpenSesame 3.1 (osdoc.cogsci.nl)^73^ and custom scripts.

### Heart rate variability

HR was continuously recorded using the V800 HR monitor (Polar Electro Italia Srl, Bologna, Italy), a mobile HR monitor that has been shown to record changes in consecutive heart beats as accurate as conventional HR monitors^74^. The V800 comprises a two-lead chest belt system (HRM strap – Polar H7) for data recording (sample rate of 1000 Hz) and a wristwatch for data storage. Device specific software (Polar FlowSync; flow.polar.com/start) was used to synchronise the recorded data from the wristwatch to Polar Flow web platform (flow.polar.com) from which HR recordings were downloaded to a computer for data processing with Kubios HRV analysis package 3.1 (www.kubios.com)^75^. According to established guidelines^76^, the HR recordings were firstly detrended (smooth priors: λ = 500), visually inspected and, when necessary, artefact corrected using adaptive filtering (cubic spline interpolation). Following artefact correction, the HR recordings were subjected to both temporal and spectral analysis to estimate different vmHRV measures. As for the temporal analysis, the root mean square of successive differences (RMSSD), measured in milliseconds, that is considered a stable^77^ and valid time-domain measure of vmHRV^54,78^, was extracted for each participant. As for the spectral analysis, the Burg autoregressive modeling based method^75^ (AR model-order = 16; for details see^79,80^) was used to estimate high-frequency HRV (HF-HRV, 0.15–0.4 Hz) power, low-frequency HRV (LF-HRV, 0.04–0.15 Hz) power, as well as high and low frequency normalized powers (HF-HRV n.u., LF-HRV n.u.) for each participant. In particular, the normalized units represent the relative value of HF- and LF-HRV power components in proportion to the total power minus the very low frequency (VLF-HRV, 0.003–0.04 Hz) component^54^ and were obtained from the absolute values according to the formulae:

$$HF \mbox{-} HRV\,n.\,u.\,=\,HF[m{s}^{2}]/(total\,power[m{s}^{2}]-VLF[m{s}^{2})$$

$$LF \mbox{-} HRV\,n.\,u.\,=\,LF[m{s}^{2}]/(total\,power[m{s}^{2}]-VLF[m{s}^{2})$$

HF-HRV and LF-HRV values were natural log transformed (namely, Log-HF-HRV and Log-LF-HRV) to fit assumptions of linear analyses^81^. Among all the frequency-domain measures obtained the HF-HRV and HF-HRV n.u. powers are considered to represent vmHRV and were used as frequency-domain measures of vmHRV. Additionally, a measure of respiration frequency, named peak high-frequency heart rate variability (pHF-HRV) values were obtained from autoregressive analyses to control for potential respiratory-induced bias^32,53^. Thus, taking into account the above considerations, in this study, the RMSSD, the HF-HRV and HF-HRV n.u. were considered as the primary indices of the self-regulatory effort needed for the explicit judgment of the video-clips.

### Behavioral and physiological analyses

RTs smaller than 300 ms were considered as outliers and discharged from the following analyses, the overall distribution of the RTs was then compared with a standard normal distribution according to an ad-hoc created routine in R (the R script can be obtained by emailing MB). RTs and scores per each block were considered in behavioral analyses. To identify vmHRV outliers based on resting stress level values, after artifact correction, the 5-min resting HR recordings were analysed to assess the Sympathovagal Balance Index (SVI)^62–64^. The SVI was computed according to the formula^62–64^:

$$SVI=\frac{LF-HRV\,power}{HF-HRV\,power}$$

After the calculation of participants’ SVI, the oulier labeling rule^82,83^ was used to identify and exclude possible outliers in our population based on resting SVI values (see Supplementary Information for further details about SVI outliers detection). Taking into account that several works showed that vmHRV changes can be reliably computed using ultra-short-term recordings (UST; less than 5-min of duration)^84–86^, we relied on an UST time-window of 110 s, on average, to obtain a fine-grained vmHRV measure capable of detecting the rapid changes in autonomic responses associated with DEAR effects. Thus, the HR recordings were divided into 8 sample-windows of 110 s each to detect the physiological response associated with each experimental condition. As a consequence, for each subject, we obtained 8 values for each vmHRV measures considered in the analyses, namely, RMSSD, HF-HRV and HF-HRV n.u.

### Statistics

All the analyses were run in the R-studio (version: 1.1.442) environment using ad-hoc created routines (the R script can be obtained by emailing MB) based on the standard libraries available online. Preliminary analyses were performed to investigate the characteristics of male and female participants. To this end, a series of Student’s *t*-test for independent samples were used to compute differences in age (years), Body Mass Index (BMI, kg/m^2^), Resting RMSSD, Resting Log-HF-HRV, and Resting HF-HRV n.u., Resting pHF-HRV and Resting SVI between male and female participants. The same analyses were performed to investigate Resting LF-HRV, as well as Resting LF-HRV n.u. (data not shown for details see Supplementary Information, see Table S-4). A further student’s *t*-test was used to explore the IAT D score used to asses the implicit racial bias of participants, while a series of one-sample Wilcoxon test were used to explore other behavioural scales (namely IMS, EMS, EES, Subtle and Blatant Prejudice Scales and TES). In order to assess stimuli- and race-related differences in behavioural data (namely, Scores and RTs) collected during both single and dual-task experimental conditions, we estimated a mixed model (‘lme4’ R package^87^, version: 1.1–17) with Type of stimuli (Harmful vs. Neutral) and Race (Africans vs. Caucasians) as fixed predictors while the Subject was considered as clustering factor to model random intercept and the type of stimuli were used to model random slope (see Supplementary Information for further details about the model’s syntax). The model with the best fit to the data was selected on the basis of likelihood ratio tests and goodness of fit indexes^88^, moreover, if significant, the Type of stimuli-by-Race interaction effect was further explored by means of pairwise comparisons while adopting an FDR correction of multiple comparisons.

To assess the existence of vmHRV differences during the explicit judgment of painful experience between African and Caucasian actors, the RMSSD, HF-HRV and HF-HRV n.u. values obtained in both single and dual-task experimental conditions were entered as dependent variables into a mixed model with Type of stimuli (Painful vs. Neutral) and Race (Africans vs. Caucasians) as fixed predictors with random intercept (grouped by subject) and random slope (grouped by Type of stimuli). If significant, the Type of stimuli-by-Race interaction effect was further explored by means of pairwise comparisons while adopting an FDR correction of multiple comparisons (see Supplementary Information for further details about the model’s syntax).

We then explored the relationship between resting vmHRV measures and RTs in the single-task condition. Differences between RTs elicited by African vs. Caucasian painful stimulations (i.e. the behavioural cost associated with judging the painful experience of African actors), were computed according to the following formula:

$${\rm{\Delta }} \mbox{-} RT \mbox{-} AC=RTs\,African\,painful\,stimulation\,-\,RTs\,Caucasian\,painful\,stimulation$$

A simple regression analysis was used to test the association between Δ-RT-AC and resting RMSSD, HF-HRV and HF-HRV n.u. values (see Supplementary Information for further details about the model’s syntax).

A similar approach was adopted to explore the relationship between vmHRV mesaures, calculated during the single-task condition, with the subjective level of implicit racial bias (IAT). In particular, at first, differences between RMSSD, HF-HRV and HF-HRV n.u. values for painful vs. neutral conditions were calculated both in Africans (vmHRV-A) and Caucasians (vmHRV-C) using the following formulae:

$$vmHRV \mbox{-} A=vmHRV \mbox{-} mesaure \mbox{-} African\,painful\,-\,vmHRV \mbox{-} mesaure \mbox{-} African\,neutral;$$

$$vmHRV \mbox{-} C=vmHRV \mbox{-} measure \mbox{-} Caucasian\,painful\,-\,vmHRV \mbox{-} measure \mbox{-} Caucasian\,neutral$$

Subsequently, the vmHRV-A (namely, RMSSD-A, HF-HRV-A and HF-HRV n.u.-A) were subtracted to vmHRV-C (by obtaining: Δ-RMSSD-AC, Δ-HF-HRV-AC and Δ-HF-HRV n.u.-AC) using the following formula,

$${\Delta } \mbox{-} vmHRV \mbox{-} AC=vmHRV \mbox{-} A\,-\,vmHRV \mbox{-} C$$

The Δ-RMSSD-AC, Δ-HF-HRV-AC and Δ-HF-HRV n.u.-AC values, that according to our theoretical model should reflect the physiological counterpart of the out-group DEAR effect, were then included in a simple regression analysis to test their association with the IAT D score (see Supplementary Information for further details about the model’s syntax).

## Supplementary information

Supplementary Information

## Data Availability

Data reported in this manuscript are available on request from the authors.
