# Supplementary material for: Heart Rate Variability reveals the fight between racially biased and politically correct behaviour
Source: Sci Rep. 2019 Aug 8;9:11532. doi: 10.1038/s41598-019-47888-w (PMC6687825; doi:10.1038/s41598-019-47888-w)
Supplement: Supplementary file 1 — Supplementary Information [file 41598_2019_47888_MOESM1_ESM.docx]

**Supplementary Information**

**Heart Rate Variability reveals the fight between racially biased and politically correct behaviour**

Di Palma M.^1^, Arcangeli E. ^1^, Lattanzi D.^2^, Gabbiadini A.^3^, Gallucci M.^3, 4^, Cuppini R.^2^, Minelli A.^2*^, Berlingeri M.^1, 4, 5*^

^1^ Department of Humanistic Studies, University of Urbino Carlo Bo, Urbino, Italy

^2^ Department of Biomolecular Sciences, University of Urbino Carlo Bo, Urbino, Italy

^3^ Department of Psychology, University of Milano-Bicocca, Milano, Italy

^4^ NeuroMi, Milan Centre for Neuroscience, Milan, Italy

^5^ Center of Developmental Neuropsychology, Area vasta 1, ASUR Marche, Pesaro, Italy

*Corresponding: andrea.minelli@uniurb.it, manuela.berlingeri@uniurb.it

**Supplementary Methods**

According to established recommendations for using HRV in psychophysiological research^1^, an ad-hoc created questionnaire was administered to all participants at the beginning of the experimental session. The ad-hoc created questionnaire included items associated with different variables that may affect the HRV recordings and results (see Fig. S-1).

*
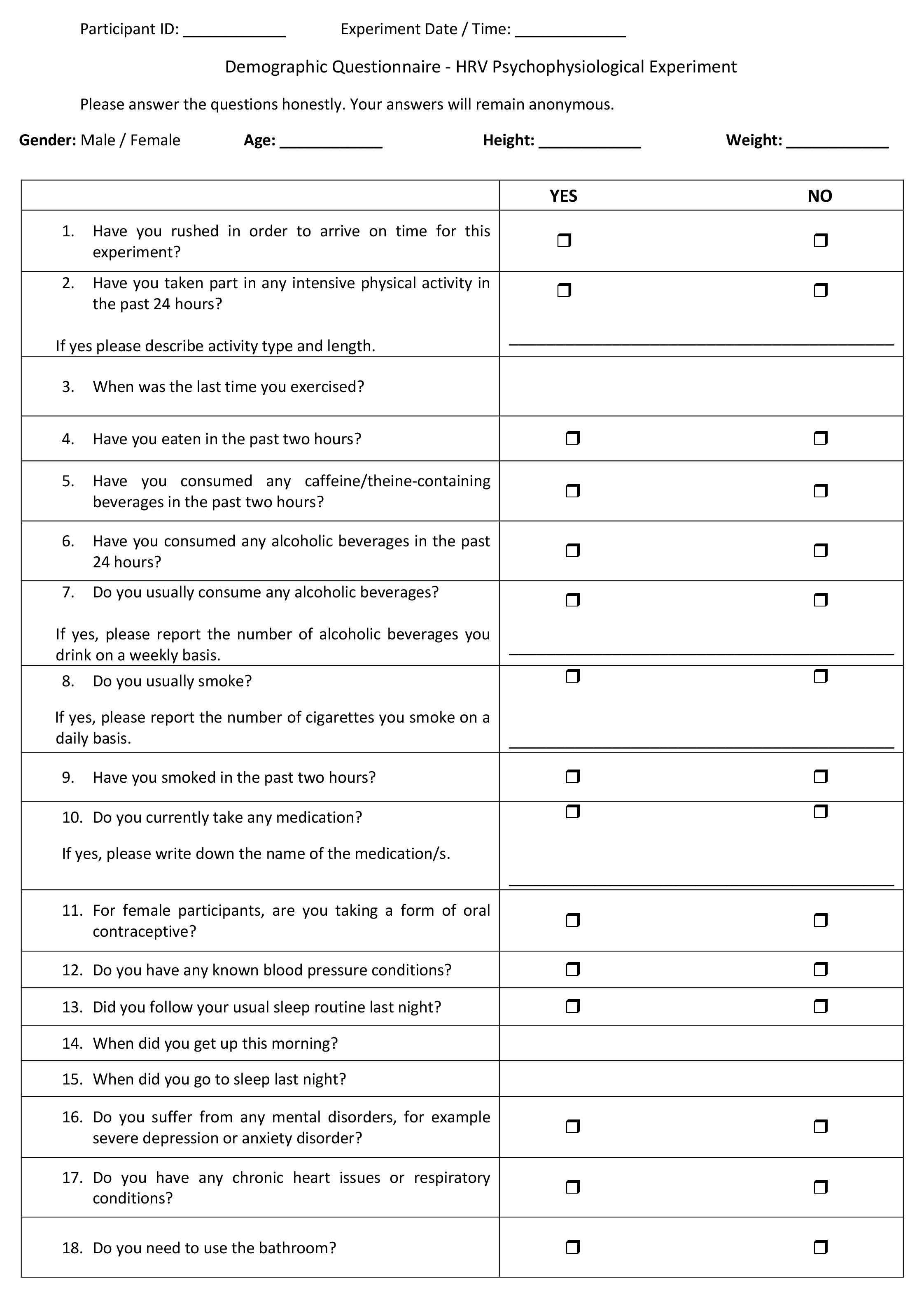
*

**Supplementary Figure 1.** The ad-hoc created questionnaire administered to all participants at the beginning of the experimental session.

**The Sympathovagal Balance Index calculation and identification of the resting high-stress outliers.** To identify HRV outliers based on resting stress level values, the 5-min resting HR recordings were analysed to assess the Sympathovagal Balance Index (SVI)^2-4^.

The SVI was computed according to the formula^2-4^:

$$SVI= \frac{LF-HRV power}{HF-HRV power}$$

After the calculation of all participants’ SVI, to identify and exclude possible outliers in our population based on resting SVI values, the outlier labelling rule (Tukey’s Method) was used^5,6^. In particular, this method for finding outliers uses the interquartile range to filter out very large or very small values using the following formulae:

$$Low-outliers=Fisrt quanrtile \left( Q1 \right)-g* Interquartile range \left( Q3 - Q1 \right)$$

$$High-outliers = Third quartile (Q3) + g * Interquartile range (Q3 - Q1)$$

According to the established guidelines regarding this method^5,6^, all the analyses for the detection of possible outliers were computed using a g = 2.2. Please find in the following table the descriptive statistics of resting SVI, as well as, percentiles values.

**Supplementary Table 1.** Resting SVI descriptive statistics and percentiles.

| **Resting SVI** | | | | | | | | | | | | | | |
| --- | --- | --- | --- | --- | --- | --- | --- | --- | --- | --- | --- | --- | --- | --- |
|  | | **N° of subjects** | **Median** | | **SD** | | **Min** | **Max** | | **IQR** | **Low-outliers (g=2.2)** | | **High-outliers (g=2.2)** | |
|  | | 52 | 2.23 | | 2.04 | | 0.87 | 9.70 | | 1.68 | -2.10 | | 6.99 | |
| **Percentiles** | | | | | | | | | | | | | | |
| **5** | **10** | | | **25** | | **50** | | | **75** | | | **90** | | **95** |
| 1.08 | 1.15 | | | 1.60 | | 2.24 | | | 3.29 | | | 5.89 | | 8.50 |

Following these analyses for SVI-outliers detection, four SVI-high-outliers were identified and excluded (see the following table for details).

**Supplementary Table 2.** SVI-high-outliers.

|  | **Gender** | **Age** | **SVI value** |
| --- | --- | --- | --- |
| **SVI-high-outliers** | | | |
|  | Female | 27 | 8.01 |
|  | Female | 20 | 9.70 |
|  | Female | 21 | 8.65 |
|  | Female | 25 | 8.42 |

**The model’s syntax used for behavioural and vmHRV analyses.** To assess stimuli- and race-related differences in both behavioural and psychophysiological outcomes we used a series of step-wise general linear effect models. The variables progressively involved in the model as fixed predictors were Type of stimuli (Painful vs. Neutral), and Race (Africans vs. Caucasians), while the Subject was considered as clustering factor to model random intercept. Between the Race and the Type of stimuli factors, the latter was used to model random slope. Indeed, among all the possible type of harmful or neutral stimuli, only a few stimuli were selected and for this reason, this factor was considered as a random one. On the other hand, among all the possible races we focused on the African-vs-Caucasian dichotomy. This choice was based on the results by Forgiarini et *al.*^7^ in which the physiological effect associated with the Asian-Caucasian comparison was mitigated as compared to the African-Caucasian dichotomy.

Accordingly, the basic structure of the estimated models to assess stimuli- and race-related differences in behavioural data was built using the following syntax:

*M0 = lmer (Behavioural Measure ~ (1+ Type of stimuli|Subject), data = mydata)*

*M1 = lmer (Behavioural Measure ~ Type of stimuli + (1+ Type of stimuli|Subject), data = mydata)*

*M2 = lmer (Behavioural Measure ~ Type of stimuli + Race + (1+ Type of stimuli|Subject), data = mydata)*

*M3 = lmer (Behavioural Measure ~ Type of stimuli * Race + (1+ Type of stimuli|Subject), data = mydata)*

The basic structure of the estimated models to explore HRV differences during the explicit judgment of painful experience between African and Caucasian actors was built using the following syntax:

*M0 = lmer (HRV Measure ~ (1+ Type of stimuli|Subject), data = mydata)*

*M1 = lmer (HRV Measure ~ Type of stimuli + (1+ Type of stimuli|Subject), data = mydata)*

*M2 = lmer (HRV Measure ~ Type of stimuli + Race + (1+ Type of stimuli|Subject), data = mydata)*

*M3 = lmer (HRV Measure ~ Type of stimuli * Race + (1+ Type of stimuli|Subject), data = mydata)*

**Supplementary Table 3.** Interclass Correlation Coefficient for Subjects. These indexes were adopted to identify the clustering factor that has to be considered in modelling the random intercepts. ICC = the Intraclass Correlation Coefficient, LowerCI = the Lower Confidence Interval limit, UpperCI = the Upper Confidence Interval limit, N = the total Number of individuals used in the analysis, K = the number of measurements per individual^8^.

|  | **ICC** | **LowerCI** | **UpperCI** | **N** | **k** |
| --- | --- | --- | --- | --- | --- |
| **Intraclass Correlation Coefficient** | | | | |  |
| Reaction Times – Single Task | 0.3406022 | 0.2534493 | 0.4574341 | 48 | 21.80597 |
| Reaction Times – Dual Task | 0.2087881 | 0.1416484 | 0.3085169 | 48 | 20.19434 |
| Score – Single Task | 0.141298 | 0.09031641 | 0.2217693 | 48 | 23.20532 |
| Score – Dual Task | 0.1557857 | 0.10021 | 0.2421143 | 48 | 20.98755 |
| RMSSD – Single Task | 0.8819286 | 0.8249466 | 0.925689 | 48 | 4 |
| RMSSD – Dual Task | 0.892597 | 0.840086 | 0.9326226 | 48 | 4 |
| Log-HF-HRV – Single Task | 0.8871156 | 0.8322913 | 0.9290657 | 48 | 4 |
| Log-HF-HRV – Dual Task | 0.8704251 | 0.808765 | 0.9181619 | 48 | 4 |
| HF-HRV n.u. – Single Task | 0.5856628 | 0.4503671 | 0.7133248 | 48 | 4 |
| HF-HRV n.u. – Dual Task | 0.6384014 | 0.5111664 | 0.7541609 | 48 | 4 |
| HF-HRV Peak – Single Task | 0.395361 | 0.2408146 | 0.5623297 | 48 | 4 |
| HF-HRV Peak – Dual Task | 0.1602982 | 0.1148528 | 0.2870782 | 48 | 4 |
| Log-LF-HRV – Single Task | 0.6258809 | 0.496523 | 0.7445951 | 48 | 4 |
| Log-LF-HRV – Dual Task | 0.738413 | 0.6330812 | 0.8278283 | 48 | 4 |
| LF-HRV n.u. – Single Task | 0.6197284 | 0.4893757 | 0.7398655 | 48 | 4 |
| LF-HRV n.u. – Dual Task | 0.653006 | 0.5284161 | 0.7652197 | 48 | 4 |

**The model’s syntax used for simple regressions.** The Δ-RT-AC values were included in a General Linear Model (GLM) comprising Resting HF-HRV n.u. as a continuous independent variable using the following syntax:

*M0 = glm (Δ-RT-AC ~ Resting HF-HRV n.u., data = mydata)*

Here it is worth noting that before the GLM model the Cook’s distance was computed to identify outliers (see the following figure).


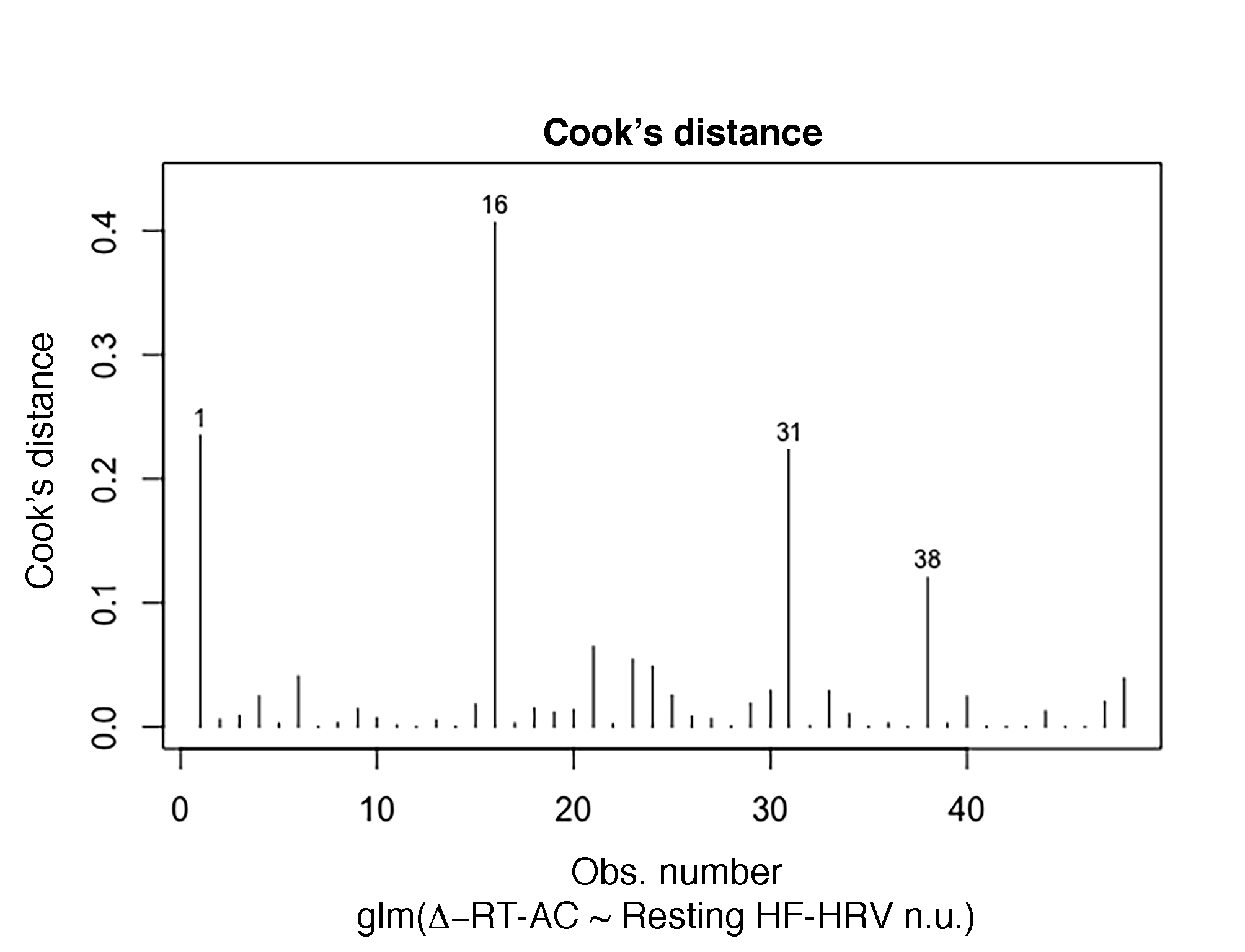


**Supplementary Figure 2.** Cook’s distance Δ-RT-AC.

The Δ-vmHRV-AC values were included in a GLM with the IAT D score as a continuous independent variable using the following syntax:

*M0 = glm (Δ-vmHRV-AC ~ IAT D Score, data = mydata)*

Here it is worth noting that before the GLM model the Cook’s distance was computed to identify outliers (see the following figure).


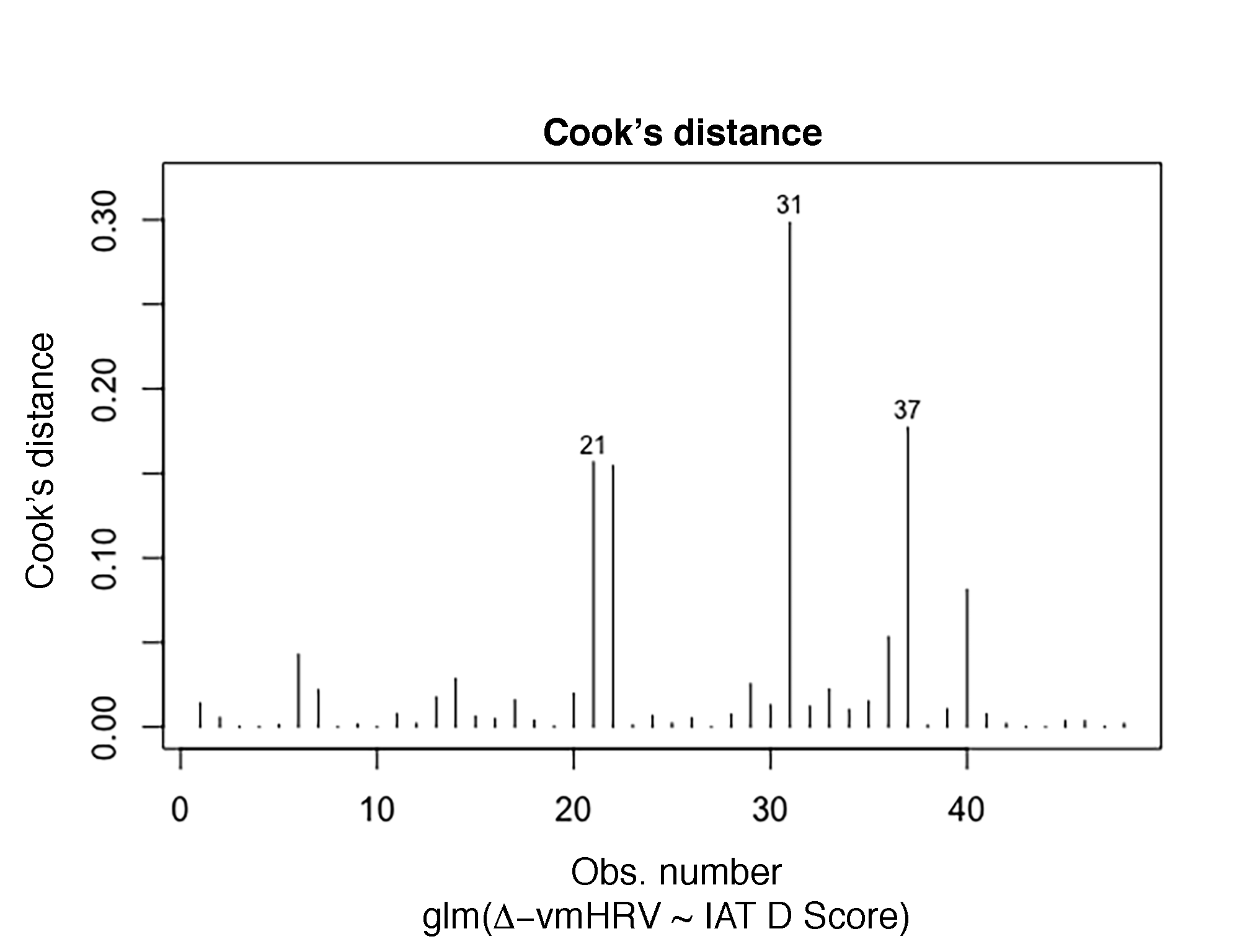


**Supplementary Figure 3.** Cook’s distance Δ-vmHRV-AC.

**Supplementary Results**

**High-Frequency Heart Rate Variability Peak values.** Prior to analyse the frequency-domain measures of vmHRV, the peak HF-HRV (pHF-HRV), a measure of respiratory frequency, was explored in both single and dual task by means of GLMM, to control for potential bias induced by respiratory frequency during the experimental tasks^9,10^. In both single and dual task experimental conditions, pHF-HRV average values were in the range of 0.15 – 0.23 Hz and no significant main effects were observed (see S-Fig. 4), thus showing that respiratory frequency did not affect the frequency-domain measures of vmHRV^1^.

**
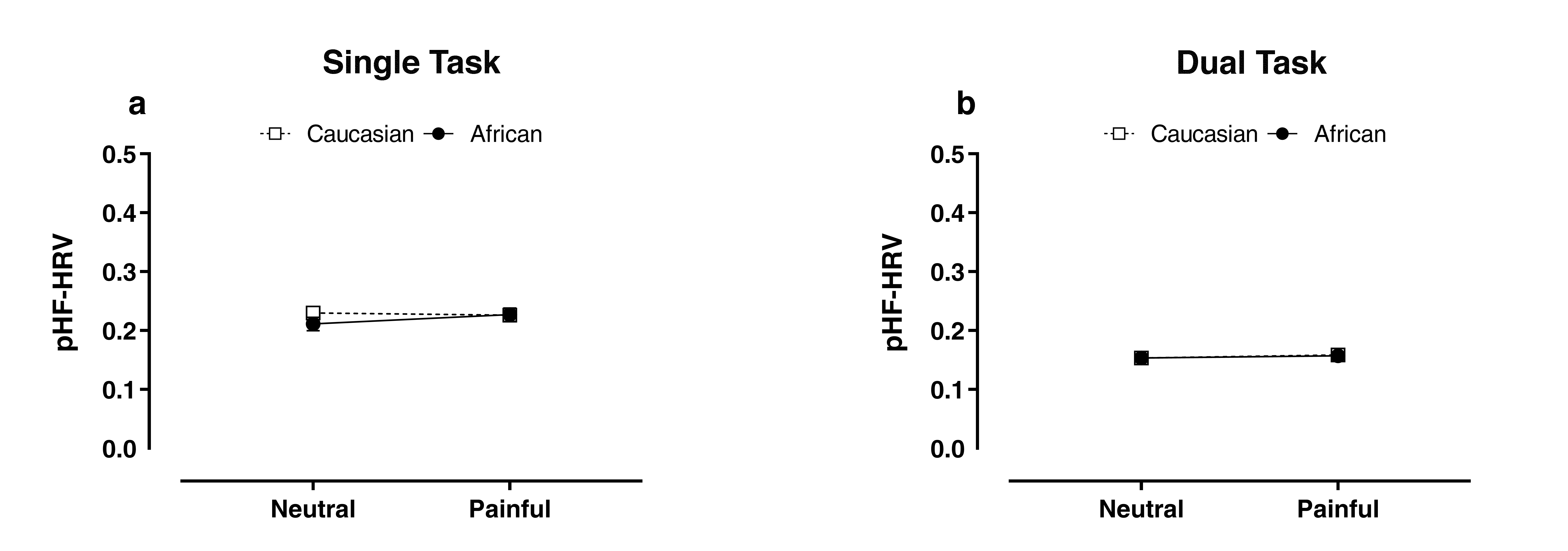
**

**Supplementary Figure 4.** pHF-HRV values collected during the experimental task. The line-plot graphs represent the pHF-HRV as a function of stimulus type and actor’s race in both the single (**a**) and dual (**b**) task conditions. Values are expressed as mean ± SEM.

**Resting Low-Frequency Heart Rate Variability.** A series of Student's *t*-test revealed no differences between male and female participants in Resting natural Log-transformed Low-Frequency power [*t_(46)_* = 1.48, *p* = .1456; see S-Table 4] and Resting Low-Frequency power normalized units [*t_(46)_* = 0.40, *p* = .6687; see S-Table 4].

**Supplementary Table 4.** Participant characteristics. Resting Log-LF-HRV = natural log transformed low-frequency heart rate variability power from HR resting recordings (5-min) and Resting LF-HRV n.u. = low-frequency heart rate variability power normalized units from HR resting recordings (5-min).

|  | **Male participants** | | | | **Female participants** | | | | **Test statistic** | |
| --- | --- | --- | --- | --- | --- | --- | --- | --- | --- | --- |
|  | **M** | **SEM** | **Min** | **Max** | **M** | **SEM** | **Min** | **Max** | ***t*, *df*** | ***p*** |
| Resting Log-HF-HRV | 7.49 | 0.15 | 5.87 | 9.07 | 7.15 | 0.16 | 5.30 | 8.30 | 1.48, 46 | 0.1456 |
| Resting LF-HRV n.u. | 68.92 | 1.74 | 46.50 | 83.90 | 67.80 | 1.94 | 52.95 | 84.34 | 0.40, 46 | 0.6687 |

**Low-Frequency Heart Rate Variability.** The pattern of results emerged from LF-HRV analyses mirrored the one obtained with HF-HRV (see Fig. 3b, c, e and f). In particular, with Log-LF-HRV in the single task condition we found a significant *type of stimuli-by-race interaction effect* (*χ^2^* _(1,192)_ = 6.29; *p* = .012). This interaction was further explored, and we observed a higher Log-LF-HRV referred to Caucasian painful stimulation - thus a significant race effect - (*χ^2^* _(1,192)_ = 5.51; *p_FDR-corrected_* = .03) and a significant type of stimuli effect for African actors only (*χ^2^* _(1,192)_ = 6.33; *p_FDR-corrected_* = .02; see Fig. S-5a). For the LF-HRV n.u. in the single task condition, we found a significant *type of stimuli-by-race interaction effect* (*χ^2^* _(1,192)_ = 6.29; *p* = .012) with a higher LF-HRV n.u. referred to Caucasian painful stimulation (*χ^2^* _(1,192)_ = 5.18; *p_FDR-corrected_* = .04) and a significant type of stimuli effect specific for Africans actors (*χ^2^* _(1,192)_ = 7.29; *p_FDR-corrected_* = .01; see Fig. S-5b). On the other hand, in the dual-task condition, no significant main effects were observed (see Fig. S-5c and d).

**
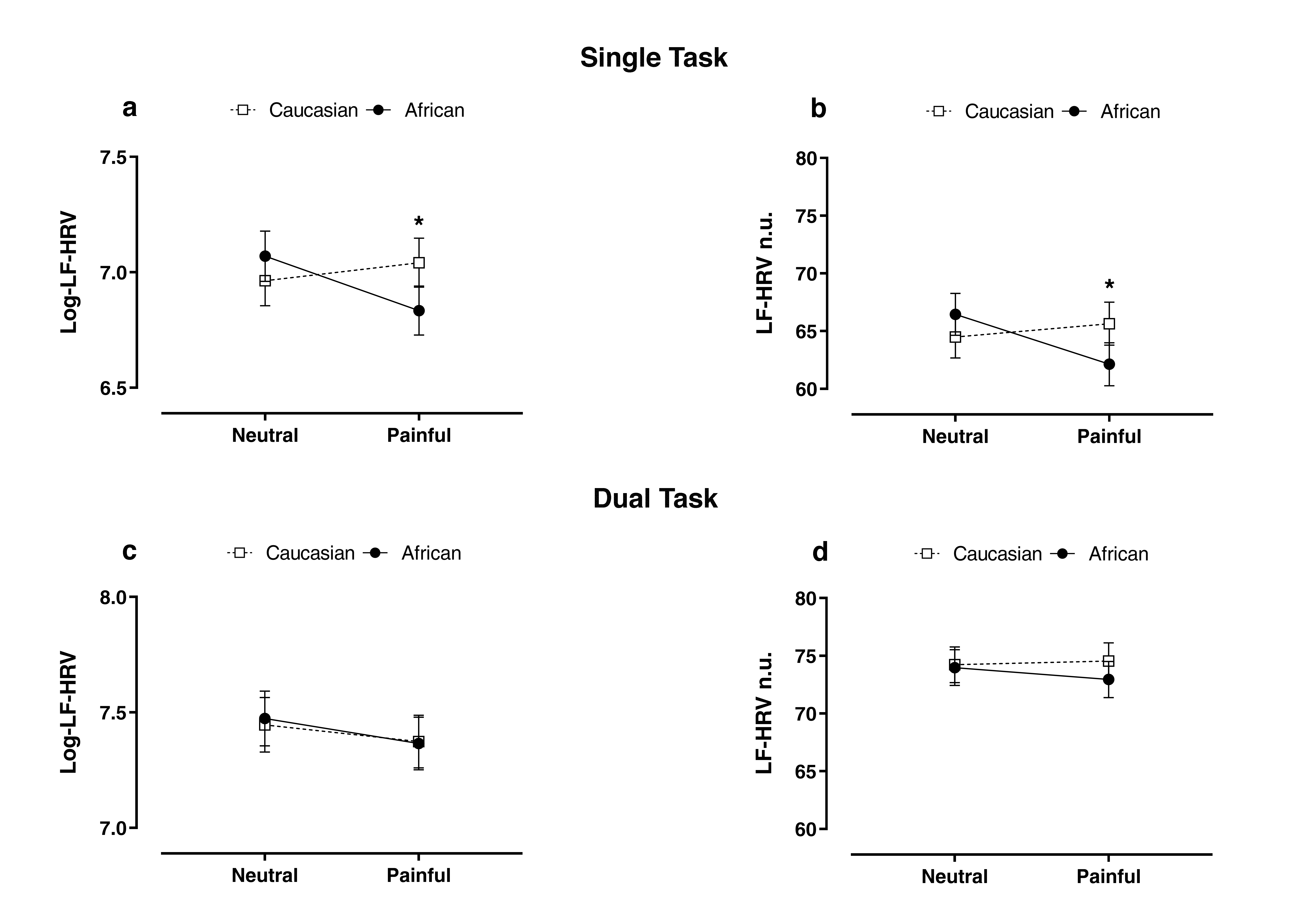
**

**Supplementary Figure 5.** LF-HRV differences collected during the experimental task. The line-plot graphs represent the Log-LF-HRV and LF-HRV n.u. as a function of stimulus type and actor’s race in both the single (**a** and **b**) and dual (**c** and **d**) task conditions. Values are expressed as mean ± SEM; **p* < 0.05.

**Supplementary References**

1 Laborde, S., Mosley, E. & Thayer, J. F. Heart Rate Variability and Cardiac Vagal Tone in Psychophysiological Research – Recommendations for Experiment Planning, Data Analysis, and Data Reporting. *Frontiers in psychology* **8**, doi:10.3389/fpsyg.2017.00213 (2017).

2 Kumar, M., Weippert, M., Vilbrandt, R., Kreuzfeld, S. & Stoll, R. Fuzzy Evaluation of Heart Rate Signals for Mental Stress Assessment. *IEEE Transactions on Fuzzy Systems* **15**, 791-808, doi:10.1109/TFUZZ.2006.889825 (2007).

3 Taelman, J., Vandeput, S., Spaepen, A. & Van Huffel, S. in *4th European Conference of the International Federation for Medical and Biological Engineering.* (eds Jos Vander Sloten, Pascal Verdonck, Marc Nyssen, & Jens Haueisen) 1366-1369 (Springer Berlin Heidelberg).

4 Boonnithi, S. & Phongsuphap, S. in *2011 Computing in Cardiology.* 85-88.

5 Hoaglin, D. C., Iglewicz, B. & Tukey, J. W. Performance of Some Resistant Rules for Outlier Labeling. *Journal of the American Statistical Association* **81**, 991-999, doi:10.1080/01621459.1986.10478363 (1986).

6 Hoaglin, D. C. & Iglewicz, B. Fine-Tuning Some Resistant Rules for Outlier Labeling. *Journal of the American Statistical Association* **82**, 1147-1149, doi:10.1080/01621459.1987.10478551 (1987).

7 Forgiarini, M., Gallucci, M. & Maravita, A. Racism and the empathy for pain on our skin. *Frontiers in psychology* **2**, 108, doi:10.3389/fpsyg.2011.00108 (2011).

8 Lessells, C. M. & Boag, P. T. Unrepeatable Repeatabilities: A Common Mistake. *The Auk: Ornithological Advances* **104**, 116-121, doi:10.2307/4087240 %J The Auk: Ornithological Advances (1987).

9 Berntson, G. G. *et al.* Heart rate variability: Origins methods, and interpretive caveats. *Psychophysiology* **34**, 623-648, doi:10.1111/j.1469-8986.1997.tb02140.x (1997).

10 Thayer, J. F., Sollers, J. J., Ruiz-Padial, E. & Vila, J. Estimating respiratory frequency from autoregressive spectral analysis of heart period. *IEEE Engineering in Medicine and Biology Magazine* **21**, 41-45, doi:10.1109/MEMB.2002.1032638 (2002).
